# Seroprevalence of SARS-CoV-2 infection among children in Children’s Hospital Zagreb during the initial and second wave of COVID-19 pandemic in Croatia

**DOI:** 10.11613/BM.2021.020706

**Published:** 2021-04-15

**Authors:** Jasna Lenicek Krleza, Renata Zrinski Topic, Vladimir Stevanovic, Amarela Lukic-Grlic, Irena Tabain, Zrinjka Misak, Goran Roic, Bernard Kaic, Dijana Mayer, Zeljka Hruskar, Ljubo Barbic, Tatjana Vilibic-Cavlek

**Affiliations:** 1 Department of Laboratory Diagnostics, Children’s Hospital Zagreb, Zagreb, Croatia; 2 Department of Microbiology and Infectious Diseases with Clinic, Faculty of Veterinary Medicine, University of Zagreb, Zagreb, Croatia; 3 School of Medicine, University of Zagreb, Zagreb, Croatia; 4 Department of Virology, Croatian Institute of Public Health, Zagreb, Croatia; 5 Department of Pediatrics, Children’s Hospital Zagreb, Zagreb, Croatia; 6 Department of Pediatric Radiology, Children’s Hospital Zagreb, Zagreb, Croatia; 7 Department of Epidemiology, Croatian Institute of Public Health, Zagreb, Croatia; 8 Department for Monitoring and Improving of School and Youth Health, Croatian Institute of Public Health, Zagreb, Croatia

**Keywords:** COVID-19, SARS-CoV-2, anti-SARS-CoV-2 antibodies, children, seroprevalence

## Abstract

**Introduction:**

The study aimed to investigate the prevalence and titres of anti-SARS-CoV-2 antibodies in children treated at the Children’s Hospital Zagreb in the first and the second wave of the COVID-19 pandemic. Statistical significance of difference at two time points was done to determine how restrictive epidemiological measures and exposure of children to COVID-19 infection affect this prevalence in different age groups.

**Materials and methods:**

At the first time point (13th to 29th May 2020), 240 samples and in second time point (24th October to 23rd November 2020), 308 serum samples were tested for anti-SARS-CoV-2 antibodies by enzyme-linked immunosorbent assay (ELISA) and electrochemiluminescence immunoassay (ECLIA). Confirmation of results and titre determination was done using virus micro-neutralization test. Subjects were divided according to gender, age and epidemiological history.

**Results:**

Seroprevalence of anti-SARS-CoV-2 antibodies differs significantly in two time points (P = 0.010). In first time point 2.9% of seropositive children were determined and in second time point 8.4%. Statistically significant difference (P = 0.007) of seroprevalence between two time points was found only in a group of children aged 11-19 years. At the first time point, all seropositive children were asymptomatic with titre < 8. At the second time point, 69.2% seropositive children were asymptomatic with titre ≥ 8.

**Conclusions:**

The prevalence of anti-SARS-CoV-2 antibodies was significantly lower at the first time point than at the second time point. Values of virus micro-neutralization test showed that low titre in asymptomatic children was not protective at the first time point but in second time point all seropositive children had protective titre of anti-SARS-CoV-2 antibodies.

## Introduction

In December 2019, a novel coronavirus emerged in Wuhan, China (*1*). The virus was named severe acute respiratory syndrome coronavirus 2 (SARS-CoV-2) and disease COVID-19. Due to a rapid spread and possibility of causing severe and life-threatening infections, it has attracted worldwide attention. On 11^th^ March 2020, the World Health Organization (WHO) declared a pandemic of the COVID-19 (*2*). On the same date, the Croatian Minister of Health declared an epidemic of COVID-19. In Croatia, as in many other countries in the world, restrictive epidemiological measures were introduced (initial lockdown) to prevent the spreading of the disease and to get the time to reorganize the entire healthcare system accordingly (*3*). With the gradual relaxing of restrictive measures in May and during the summer, the number of new COVID-19 cases gradually increased daily, but in September and October, this number dramatically and exponentially increased in many countries, especially in Europe. It was difficult to talk about the new (or the second) wave of epidemic since the virus had not disappeared at any moment and practically the first wave has not “finished”. However, many states introduced new different restrictive measures in September and October 2020 due to the “new” wave of COVID-19. In Croatia, despite a significant daily increase in the number of new cases, new restrictive measures were introduced in late November (*4*).

Incidence data of COVID-19 in the paediatric population are available from epidemiological reports of countries with the highest number of cases. Reports show a small proportion of children (0-19 years) in the total number of patients. In China, children under 18 amounted to 2-5%, Italy 1.2%, United States 7.3%, and in Australia 4% of all COVID-19 positive cases (*5*-*9*). The first multinational and multicentre study on children with COVID-19 in Europe during the initial peak of the pandemic, which was conducted in 82 tertiary and quaternary paediatric units in 25 European countries, showed that COVID-19 is generally a mild disease in children, including infants, and a proportion of 8% of those COVID-19 positive children developed a severe illness that required intensive care support and prolonged mechanical ventilation. Several predisposing factors for intensive care support have been identified, and it is confirmed that death is rare in children (*10*).

All these data suggest that children show clinical symptoms less often than adults, and they also have a milder illness, recover faster and have a better prognosis. The role of asymptomatic or subclinical infection in human-to-human transmission of the virus is not fully understood.

Due to mild or asymptomatic infections, children are not included in the routine molecular testing (reverse transcriptase polymerase chain reaction; RT-PCR) for COVID-19 and according to existing data, it is impossible to accurately determine the number of infected children (*11*). Generally, mildly affected or asymptomatic persons are not routinely tested and included in the COVID-19 reports and the number of infections is probably underestimated. In this context, seroprevalence studies are important in the assessment of the extent of infection in the population. As the WHO recommended, monitoring changes in the seroprevalence over time is also crucial to predict dynamics and plan adequate public health measures (*12*).

Testing for specific antibodies has far greater potential than molecular testing to detect a past asymptomatic patients or patients with mild symptoms of infection. Antibodies most commonly become detectable 1-3 weeks after symptom onset, at which time evidence suggests that infectiousness likely is greatly decreased and that some degree of immunity has developed (*13*). Existing commercial assays generally detect SARS-CoV-2 immunoglobulin (Ig)A, IgM and IgG class antibodies separately or total antibodies specific for the nucleocapsid or spike protein of the virus (*11*).

Children’s Hospital Zagreb (CHZ) is a general hospital for children aged 0 to 19 years whose primary role is not the treatment of COVID-19 and the aim of this study was to investigate the prevalence and titres of anti-SARS-CoV-2 antibodies in children treated at the CHZ in the first and the second wave of the COVID-19 pandemic. A statistical significance of the difference of anti-SARS-CoV-2 prevalence at two time points was done to determine how restrictive epidemiological measures and exposure of children to COVID-19 infection affect this prevalence in children of different age groups. The results of this study represent a contribution to the assessment of the extent of COVID-19 infection in the population, but also help in monitoring seroprevalence changes to predict dynamics and planning appropriate public health measures in Croatian pandemic conditions.

## Materials and methods

### Study design and subjects

All blood samples of children which arrived at the Department of Laboratory Diagnostics of CHZ that met the residual criterion volume (> 350 µL) after the routine laboratory analysis were included in the study at two time points of COVID-19 pandemic. The first time point was from 13^th^ to 29^th^ May 2020 (just after the lockdown, number of COVID-19 positive patients was 0 to 8 *per* day at the national level), and in the second time point from 24^th^ October to 23^rd^ November 2020 (the peak of the second wave of the COVID-19 pandemic, until new restrictive measures were introduced, the number of COVID-19 positive patients was 1165 - 3573 *per* day at the national level).

Samples collected during the first and second time points came from independent groups of subjects. During the first time point, the number of subjects was defined according to the protocol of multi-center study of anti-SARS-CoV-2 antibodies seroprevalence in the general population where 240 samples were collected in CHZ and tested in Croatian Institute of Public Health (CIPH). At second time point, the CHZ proposed, collected, and determined the samples collected during one month at the peak of the second wave (N = 308).

All subjects were divided according to gender and age into three groups: (i) < 1 year; (ii) 1-10 years and (iii) 11-19 years. The reason for this age-based classification is the exposure of children to the SARS-CoV-2 virus, which varies according to the age of the child. The assumption is that the newborns and infants are mostly at home with their families, while older children are mostly exposed to the virus in kindergarten and school. In addition to the school exposure, teenagers are exposed due to a more intense social life.

According to the epidemiological history, the subjects were classified as (i) negative, (ii) potentially positive and (iii) positive for COVID-19. Table 1 shows the criteria for distribution to groups of children according to epidemiological history.

**Table 1:** Distribution of children according to epidemiological history and/or COVID-19 test

**Epidemiological history**		
**Negative** **(all criteria)**	**Potentially positive** **(one or more criteria)**	**Positive** **(one of criteria)**
Children who have not been outside Croatia in the last 14 days	Children who stayed outside Croatia in the last 14 days	Children recovered from COVID-19 (< 2 months)
Children who have not been in contact with COVID-19 patients for the past 14 days	Children who have been in contact with COVID-19 positive patients for the past 14 days	Children with positive COVID-19 test (positive RT-PCR test within 24 hours)
Children without a fever	Children with fever	
Children with fever, but not respiratory symptoms	Children with fever and respiratory, gastrointestinal or neurological symptoms	
Children with negative COVID-19 RT-PCR test presented to the hospital for other reasons	Children who should be admitted to hospital for other reasons, until the results of the RT-PCR test	
RT-PCR - Reverse transcription polymerase chain reaction.		

Samples were taken in the laboratory facilities (outpatients), emergency department (children with acute disease), isolation department (children to be admitted to hospital, prior to the results of the reverse transcription polymerase chain reaction (RT-PCR) test), other hospital department (hospitalized patients), and daily hospital or specialized pediatric clinics (follow-up examinations without active disease). According to the medical history, diagnoses of subjects included: ulcerative colitis, irregular menstruation, obstructive defects of the renal calyx and urethra, conditions following recovery from infectious mononucleosis, bone fractures, cystic fibrosis, appendicitis, fever of unknown origin, Crohn’s disease, dermatitis, epilepsy, anaemia, pyelonephritis, asthma as well as children with solid tumors and leukemia. Symptoms at the time of ant-SARS-CoV-2 antibodies determination are grouped and listed in Table 2. Potentially positive children and children suspected for SARS-CoV-2 infection were not considered as an exclusion criteria in order for the level of infection in the general population would not be underestimated.

**Table 2:** Clinical characteristics of children with the positive anti-SARS-CoV-2 test

	**Initial „first“ wave** **(13^th^ to 29^th^ May, 2020)** **N = 240**	**Second wave** **(24^th^ October to 23^rd^ November)** **N = 308**
Number (%) of children with positive anti-SARS-CoV-2 and positive mVNT	7 (2.9)	26 (8.4)
COVID-19 RT-PCR positive	0/7	7/26
Acute disease	0/7	7/26
Chronic disease	7/7	6/26
Healthy (follow-up examinations without active disease)	7/7	13/26
Hospitalized	0/7	6/26
Emergency hospital admission	0/7	4/26
Daily hospital	4/7	7/26
Outpatient	3/7	9/26
Symptoms at the time of anti-SARS-CoV-2 determination		
Fever (> 38 °C) with or without other symptoms	0	2
Respiratory symptoms (cough, runny nose, dyspnoea)	0	0
Neurological symptoms (headache, dizziness, convulsions)	3*	0 (1*)
Urological symptoms (urinary tract infection, renal colic)	2*	2
Gastrointestinal symptoms (nausea, vomiting, diarrhoea, loss of appetite)	1*	4 (5*)
Other unspecified symptoms (abdominal pain or chest pain)	1*	4
Asymptomatic	7/7	18/26
mVNT - virus micro-neutralization test. RT-PCR - Reverse transcription polymerase chain reaction. *symptoms of the underlying disease.		

Informed consent was obtained from parents of all children included in the study. CIPH and CHZ Ethics Committee approved this study.

### Blood sampling

Patient’s venous blood was collected in a tube with clot activator and gel separator (Vacuette, Greiner Bio-One GmbH, Austria). Centrifugation of the blood samples was applied according to the manufacturer’s recommendation (2000xg, 10 minutes), the samples were forwarded for analysis and immediately after analysis (maximum 4 hours after centrifugation) the remaining serum was separated into labelled plastic tubes and stored at - 20 °C until analysis (maximum 3 weeks).

Blood sampling for outpatients were performed in fasting state in the morning from 8 to 10 am, but for day hospital and hospital departments, patient’s blood samples were collected throughout the day in fasting and non-fasting state. Therefore, all lipaemic samples were not included in this study. In addition, haemolysed and icteric samples were also excluded from the study.

### Methods

Serological testing during the first and second waves of the COVID-19 pandemic was done by different methods: enzyme-linked immunosorbent assay (ELISA) in the first wave at CIPH and electro-chemiluminescence immunoassay (ECLIA) in the second wave at CHZ. Both methods were verified according to the recommended protocol of American Society for Microbiology Clinical and Public Health Microbiology (*14*). The methods showed good comparability and the results of both methods were confirmed using virus micro-neutralization test (mVNT). The results of mVNT were defined as final.

#### ELISA method

A commercial ELISA (COVID-19 ELISA IgA+IgM; IgG, Vircell, Granada, Spain) was used for detection of anti-SARS-CoV-2 antibodies in human serum or plasma samples at CIPH. The assay is based on reaction to recombinant spike glycoprotein (S) and nucleocapside protein (N) antigens of SARS-CoV-2. The results were expressed as antibody index [AI = (sample optival density (OD)/ cut off serum mean OD)] x 10 and interpreted as follows: IgG < 4 negative, 4-6 borderline, > 6 positive; IgM/IgA < 6 negative, 6-8 borderline, > 8 positive (*15*). Positive, negative and controls at borderline level have been run with each test run. Initial test validation has been performed on 30 serum samples collected from patients with RT-PCR confirmed COVID-19 (N = 15) 4-34 days after disease onset and asymptomatic persons (N = 15) with negative RT-PCR and negative mVNT.

#### ECLIA method

Roche Cobas Elecsys Anti-SARS-CoV-2 is an ECLIA test for qualitative detection of IgG and IgM class of antibodies (multiple analytes reported as a single result) developed against anti-SARS-CoV-2 antibodies in human plasma or serum samples on Cobas e-411 immunoassay analysers (Roche Diagnostics GmbH, Mannheim, Germany) at CHZ. The assay is based on a recombinant protein which represents the nucleocapsid (N) antigen of SARS-CoV-2.

The result of sample testing is given either as reactive or non-reactive as well as in the form of a cut-off index (COI; signal sample/cut-off). Samples results with COI < 1.0 is negative for anti-SARS-CoV-2 antibodies and COI ≥ 1.0 is positive for anti-SARS-CoV-2 antibodies. Verification of the ECLIA method was done according to the American Society for Microbiology recommendations and protocol (*14*).

The method verification procedure as well as the testing of the collected samples used the original commercial reagent with the original respective calibrators. Control samples for determining precision and accuracy and as an internal control in each series of sample run used PreciControl Anti SARS-CoV-2 control (Roche Diagnostics GmbH, Mannheim, Germany) in two levels (Level 1: range 0.00 - 0.80 COI, mean: 0.40 COI and Level 2: range 1.73 - 4.64 COI and mean 3.20 COI).

Verification ECLIA method in CHZ was done at the first time point and parallel serum samples were separated (N = 50) and stored for comparability of results by ECLIA method testing (CHZ) with results by ELISA method testing (CIPH). Verification showed that the method is comparable to ELISA and gives acceptable identical results (35/36 same results, data not published).

#### Virus micro-neutralization test

All reactive samples (by ELISA and/or ECLIA method) were confirmed using a virus mVNT in cell culture in biosafety level 3 (BSL-3) laboratory at CIPH. The SARS-CoV-2 HR1/8933 strain isolated from the nasopharyngeal swab of COVID-19 patient on Vero E6 cells was used for the mVNT. Maximum cytopathic effect was visible on the 4th day and the virus replication was confirmed by RT-PCR. Heat inactivated serum samples (56 °C/30 min) were tested in duplicate in 96-well plates. An equal volume (25 µL) of two-fold serum dilutions (starting from 1:2) was mixed with the equal volume (25 µL) containing 100 median tissue culture infectious doses (TCID_50_) of the virus. After 1 h incubation at 37 °C in CO_2_ incubator, 50 µL of Vero E6 cells in a concentration of 2 x 10^5^ cells/mL were added to each well and incubated for 4 days. The antibody titre was defined as the reciprocal value of the highest serum dilution that showed 100% neutralization in at least half of the infected wells. A titre of ≥ 8 was considered positive and protective and titre of 2-4 was considered positive, but unprotective (*16*).

### Statistical analysis

Results were analysed using Microsoft Excel version 2013 (Microsoft, Redmond, USA) and presented as a total number and a percentage. The age of the children for each age group was expressed as the median and range. Gender was expressed as ratio of the number of girls to the number of boys. Significance of differences of results at two time points was done by chi-square test (for N > 100) and comparison of proportions (with 95% confidence interval) (for N < 100) using MedCalc Statistical Software version 19.5.2 (MedCalc Software Ltd, Ostend, Belgium) (*17*). A statistically significant difference was defined at P < 0.05.

## Results

The first, initial wave of the pandemic was marked by a total lockdown, and samples were collected when the first strict epidemiological measures gradually mitigated. At this first study point, after mVNT, the final number of COVID-19 seropositive children was 2.9% (Table 3). The second point of the study was marked as the peak of the second wave of the pandemic, and out of 308 children samples examined, 8.4% COVID-19 seropositive children were found. Statistically significant difference was found in the number of seropositive children during the first and the second wave of the COVID-19 pandemic (P = 0.010) (Table 3). About 90% of the examined children were defined as negative according to epidemiological history at both observed time points and a significant difference during the first and second wave of the pandemic was observed only in the group of children with a positive epidemiological history (P = 0.009) (Table 3).

**Table 3:** Distribution of children according to epidemiological history and results of anti-SARS-CoV-2 and mVNT in two time points

	**Initial „first“ wave** **(13^th^ to 29^th^ May, 2020)** **N = 240**			**Second wave** **(24^th^ October to 23^rd^ November)** **N = 308**			P
Epidemiological history (at the time of sampling)	Negative	Potentially positive	Positive	Negative	Potentially positive	Positive
Number (%)	218 (90.8)	22 (9.2)	0 (0)	273 (88.7)	26 (8.4)	9 (2.9)	Negative: 0.845* Potentially positive: 0.785* Positive: 0.009*
Gender (girls/boys)	100/140			159/149			Girls: 0.164^†^ Boys: 0.199^†^
Number of positive anti-SARS-CoV-2 cases (N, %)	9 (3.8)			27 (8.8)			/
Results of mVNT	7 (with titres < 8), 2 negative			26 (25 titres ≥ 8, 1 titre < 8), 1 negative			/
Final positive results – results after mVNT (N, %)	7 (2.9)			26 (8.4)			0.010^‡^
Antibody titre (median, range)	4 (2-4)			256 (8-256)			/
Clinical specificity of test	ELISA test (CIPH): 99.2%			ECLIA test (CHZ): 99.7%			/
*Testing the significance of differences for each group of children according to epidemiological history between two time points. ^†^Testing the significance of differences for girls and boys between two time points. ^‡^Testing the significance of differences for anti-SARS-Cov-2 final positive results of children between two time points. mVNT - virus micro-neutralization test, titre ≥ 8 positive (protective) and titre < 8 (2-4) is positive but not protective. P < 0.05 was considered statistically significant. CIPH – Croatian Institute of Public Health. CHZ - Children’s Hospital Zagreb. ELISA - enzyme-linked immunosorbent assay. ECLIA - electro-chemiluminescence immunoassay.							

The distribution of results by age/gender groups showed that a statistically significant difference between seropositive children during the first and second wave of the COVID-19 pandemic is found only for the 11-19 age group (P = 0.007) (Table 4). In this age group, at the two time points, proportions of boys and girls are significantly different. Boys during the first wave is significantly higher than in the second wave (P = 0.048) in contrast to girls where proportion of girls in the first wave is significantly less than in the second wave (P = 0.044) (Table 4). Results of the distribution by gender, age, and epidemiological history are presented in Table 4.

**Table 4:** Distribution of results according to age group

	**Initial „first“ wave** **(13^th^ to 29^th^ May, 2020)** **N = 240**			**Second wave** **(24^th^ October to 23^rd^ November)** **N = 308**			**P**
**Epidemiological history** **(at the time of sampling)**	**Negative**	**Potentially positive**	**Positive**	**Negative**	**Potentially positive**	**Positive**
**Children < 1 year, N (%)**	21 (8.8)			27 (8.8)			0. 995*
N (proportion)	16 (0.76)	5 (0.24)	0 (0)	21 (0.78)	6 (0.22)	0 (0)	/
Age	6m (12d to 11m)	8m (3m to 10m)	-	8m (12d to 11m)	3m (2m 5d to 7m)	-	/
Gender, (girls/boys)	8/8	2/3	-	9/12	1/5	-	Girls: 0.639^†^ Boys: 0.706^†^
Number of positive anti-SARS-CoV-2 cases	1	1	-	2	0	-	/
Total N of positive anti-SARS-CoV-2 cases, N (proportion)	2 (0.10)			2 (0.07)			/
Results of mVNT	1 positive (titre < 8)	1 negative	-	1 positive (titre ≥ 8), 1 positive (titre < 8)	-	-	/
Final positive results – results after mVNT, N (proportion)	1 (0.05)			2 (0.07)			0.704^‡^
Antibody titre (median, range)	4			132 (8-256)			/
**Children 1-10 years, N (%)**	124 (51.7)			167 (54.2)			0.742*
N (%)	115 (92.7)	9 (7.3)	0	149 (89.2)	14 (8.4)	4 (2.4)	/
Age	5y (1y to 10y 6m)	3y (1y to 5y 2m)	-	5y (1y 1m to 10y 11m)	4y (1y 2m to 9y 3m)	7y 2m (2y 4m to 10y 11m)	/
Gender, (girls/boys)	48/67	1/8	-	74/75	9/5	1/3	Girls: 0.263^†^ Boys: 0.323^†^
Number of positive anti-SARS-CoV-2 cases	4	0	-	6	1	2	/
Total N of positive anti-SARS-CoV-2 cases, N (%)	4 (3.2)			9 (5.4)			
Results of mVNT	4 positive (titre < 8)	-	-	6 positive (titre > 8)	1 positive (titre > 8)	2 positive (titre > 8)	/
Final positive results (after mVNT), N (%)	4 (3.2)			9 (5.4)			0.398^‡^
Antibody titre (median, range)	4 (2-4)			256 (128-256)			/
**Children 11-19 years, N (%)**	95 (39.6)			114 (37.0)			0.696*
N (%)	87 (91.6)	8 (8.4)	0	103 (90.3)	6 (5.3)	5 (4.4)	/
Age	15y (11y to 18y 3m)	14y (11y 10m to 17y 10m)	-	15y (11y to 18y 8m)	15y 6m (14y 3m to 17y 7m)	16y 6m (13y 2m to 18y 7m)	/
Gender, (girls/boys)	39/48	2/6	-	59/44	3/3	3/2	Girls: 0.044^†^ Boys: 0.048^†^
Number of positive anti-SARS-CoV-2 cases	3	0	**-**	10	1	5	/
Total N of positive anti-SARS-CoV-2 cases, N (%)	3 (3.2)			16 (14.0)			/
Results of mVNT	2 positive (titre < 8), 1 negative	-	**-**	10 positive (titre > 8)	1 negative	5 positive (titre > 8)	/
Final positive results (after mVNT), N (%)	2 (2.1)			15 (13.2)			0.007^‡^
Antibody titre (median, range)	4			256 (64-256)			
*Testing the significance of differences for total number of children according age group between two time points. ^†^Testing the significance of differences for girls and boys between two time points. ^‡^Testing the significance of differences for anti-SARS-Cov-2 final positive results of children between two time points. mVNT - virus micro-neutralization test, titre ≥ 8 positive (protective) and titre < 8 (2-4) is positive but not protective. P < 0.05 was considered statistically significant. d - days. m - months. y - years.							

The share of seropositive results in group of children with negative epidemiological history during the first wave was 3.2%, and during the second wave of the pandemic it was 6.6%, with P = 0.107.

During the first wave of the pandemic, all seropositive children were asymptomatic at the time of anti-SARS-CoV-2 testing. At the second wave, 7/26 children with a positive COVID-19 RT-PCR test were found: 2 children with positive epidemiological history and positive RT-PCR (within 24 hours) and 7 children recovered from COVID-19 from 8 days to 2 months prior to testing and only 5 were seropositive.

During the second wave of the pandemic, out of a total of 26 seropositive children, 6 children were hospitalized for treatment of acute or chronic diseases, and 4 children were admitted to emergency hospital admission due to acute disease (2 children had a fever > 38 °C with other symptoms: nausea, vomiting/diarrhea, abdominal pain), and 2 children were afebrile (bone fracture, renal colic). Asymptomatic children who underwent triage before entering the day hospital for specialist examination/treatment (7 children) and before arriving at the laboratory for blood sampling (9 children) were seropositive for COVID-19. Only two seropositive children had fever > 38 °C with other symptoms (urological, gastrointestinal and/or other) and 6 children had combination of symptoms (urological, gastrointestinal and/or other) without fever and respiratory symptoms. In total, at the second wave of the pandemic, we found 18/26 seropositive asymptomatic children without the symptoms that could be associated with COVID-19 disease (Table 2).

## Discussion

The results of serological testing for COVID-19 at Children’s Hospital Zagreb show a significantly lower seroprevalence in the paediatric population during the first, initial wave, compared to the second wave of the COVID-19 pandemic (2.9% *vs.* 8.4%, P = 0.010). This significant difference was expected due to the increase of incidence in the country and the second round of seroprevalence study was conducted in order to provide evidence that the increased transmission of infection affected children as well as adults.

The first published results of COVID-19 seroprevalence from the end of March to up April 8, 2020 in Wuhan City was 9.6% in the general population and first systematic review of COVID-19 seroprevalence in May 2020 reported a very wide range of seroprevalence: from 0.4 to 59.3% in the general population (*18*, *19*).

There are few published data on the COVID-19 seroprevalence in children and mainly relate to the first wave (from April to May 2020) of the pandemic. Different results in these studies may be due to the type of the studies (the most of published data are case reports or case series), dynamics in new cases *per* day, the strength of the epidemiological measures applied in countries, and in the definition of age groups (some results are part of the general population report). However, all these data have the same conclusion that COVID-19 seroprevalence in children is low. The results of a study in Switzerland during April and May 2020 show a very low prevalence in children aged 5-9 years (0.8%) compared to children between 10 and 19 years (9.4%) (*20*). An extensive Spanish study of COVID-19 seroprevalence in the general population during April and May 2020 reported that children aged 0-19 were represented by 3.9% (*21*). US prevalence study of anti-SARS-CoV-2 antibodies in children (age range 0-18 years) without symptoms of COVID-19 disease who were tested at 28 hospitals showed the prevalence varied from 0% to 2.2%, with a pooled prevalence of 0.65% (95%CI: 0.47% to 0.83%) with significant heterogeneity and significantly associated with weekly incidence of COVID-19 in the general population (*22*). Also, the study performed at Seattle Children’s Hospital during the lockdown in March and April 2020 found only 1% of COVID-19 seropositive children aged 0 to 15 years (*23*). Multicentre observational cohort study, conducted between April to July 2020 at 5 UK sites, recruited children of healthcare workers, aged 2-16 years publish that total COVID-19 seroprevalence is 6.9% (95% CI 5.4% to 8.6%, N = 992) and varied between sites. Belfast had significantly lower seroprevalence than all other sites at 0.9% (95% CI 0.2% to 3.3%, N = 215 and P < 0.001), and in London seroprevalence was significantly higher than all other sites at 11.6% (95% CI 7.8% to 16.8%, N = 199 and P = 0.007) (*9*). In the Czech Republic, in April 2020, the overall SARS-CoV-2 seroprevalence was estimated not to exceed 1.3%. In July and August, 2020, 200 children (0 to 18 years of age) from paediatric department of a large hospital in Prague were screened for the presence of anti-SARS-CoV-2 antibodies and zero seropositive subjects were found. Therefore, this study reported a low (< 0.5%) cumulative seroprevalence amongst children in Prague during August, 2020 (*24*).

According to the initial reports (up to 29th May 2020) of the CIPH, there were 2.8% children under 10 and 4.0% children and young adults from 11 to 20 years among all COVID-19 patients in Croatia. Until November 2020, the cumulative number of children up to age 10 was 2.4%, and children and young adults ages 11 to 20 years 9.3% (*25*, *26*).

In our study, the initial, first wave, was marked by strict epidemiological measures (home-working, online schools, closed kindergartens, hospital admissions restricted). With the gradual mitigation of these strict measures in late April and early May 2020, serological testing began. The number of new RT-PCR positive cases at the national level in that period was 0 to 8. According to the literature, children are significantly less likely to suffer from COVID-19 infection, affected children show mild symptoms or are asymptomatic and children’s exposure to COVID-19 virus in the initial period was very low (*10*-*12*). Our results also show a low seroprevalence (2.9%) for children of age 0 to 19 in the first time point and all of them were asymptomatic.

The beginning of autumn was marked by a “new normal”: the opening of schools, kindergartens, health institutions, sports, social and economic institutions with certain epidemiological measures (social distance, wearing masks indoors, hand disinfection), and by the end of October the steeply upward curve of new infection reached > 3500 new cases *per* day and children’s exposure to COVID-19 infection was significantly higher. At this time point, except negative and potentially positive children according to epidemiological history, we found RT-PCR COVID-19 positive cases, as well as children recovered from COVID-19 infection. This time point being a more realistic state for determining seroprevalence in children than the initial first wave. Seroprevalence in children at the second time point was 8.4%, and 70% of them were asymptomatic.

When we distributed our subjects according to age groups, taking into account variation of epidemiological measures applied (*e.g.* infants up to 1 year exposed to COVID-19 infection almost exclusively in contact with mother and household, children staying in kindergarten or attending lower grades at primary schools with a minimum distance and mask-wearing, teenage groups attending school mostly online, and outside of-home-contacts with mandatory mask-wearing indoors by public health and social institutions and ensuring social distance), we noted that a significant difference in seroprevalence is shown exclusively in the age group of teenagers. These results refer that regardless of epidemiological measures and exposure to the COVID-19 virus, the seroprevalence of young children is low. The children are mostly asymptomatic or present a mild form of the disease which is consistent with results of other studies (*11*, *27*).

The literature cited several possible reasons related to the protective effect from severe clinical forms of COVID-19 and lethal outcomes in children: virological and epidemiological characteristics in children, the immune system and the maturation and low exposure to ACE2 receptors, characteristics of the renin angiotensin system in childhood, as well as the shorter effect of air pollutants on the respiratory system compared to the adult population (*11*, *27*, *28*). The contribution to the assumption of protective mechanisms in children is the case of one 2 months old child, the subject of this study who was considered positive according to the epidemiological history due to a COVID-19 RT-PCR positive mother at birth, but found negative for both COVID - 19 (RT-PCR) as well as anti-SARS-CoV-2 antibodies (IgM, IgG).

This study has some limitations. Although Children’s Hospital Zagreb is a specific children’s health institution in the Republic of Croatia where children from all parts of the country are treated, the obtained results of COVID-19 seroprevalence in children cannot be considered representataive at the national level.

Our results do not provide an answer on the transmission of COVID-19 disease from children to adults which should be investigated separately when planning epidemiological measures during a pandemic.

In conclusion, the prevalence of anti-SARS-CoV-2 antibodies was significantly lower at the first time point than at the second time point. Virus micro-neutralization test values showed that low titre in asymptomatic children was not protective at the first time point, but all seropositive children in the second wave had a protective titre of anti-SARS-CoV-2 antibodies. According to the results, the determination of anti-SARS-CoV-2 antibodies could be useful for children older than 10 years both in terms of their own protection and in terms of COVID-19 transmission.

Determination of mVNT in epidemiological circumstances of COVID-19 pandemic plays an important role in the verification of qualitative methods as well as their quantification by providing important epidemiological data on the protection of individuals and population. Also, for commercial quantitative tests, it will be helpful in order to define activity of anti-SARS-CoV-2 antibodies antibody as the limit of protection.
